# Data-Driven Differential Diagnosis of Dementia Using Multiclass Disease State Index Classifier

**DOI:** 10.3389/fnagi.2018.00111

**Published:** 2018-04-25

**Authors:** Antti Tolonen, Hanneke F. M. Rhodius-Meester, Marie Bruun, Juha Koikkalainen, Frederik Barkhof, Afina W. Lemstra, Teddy Koene, Philip Scheltens, Charlotte E. Teunissen, Tong Tong, Ricardo Guerrero, Andreas Schuh, Christian Ledig, Marta Baroni, Daniel Rueckert, Hilkka Soininen, Anne M. Remes, Gunhild Waldemar, Steen G. Hasselbalch, Patrizia Mecocci, Wiesje M. van der Flier, Jyrki Lötjönen

**Affiliations:** ^1^VTT Technical Research Centre of Finland, Tampere, Finland; ^2^Alzheimer Center, Department of Neurology, VU University Medical Center, Amsterdam Neuroscience, Amsterdam, Netherlands; ^3^Danish Dementia Research Centre, Rigshospitalet, Copenhagen, Denmark; ^4^Combinostics Ltd., Tampere, Finland; ^5^Institutes of Neurology and Healthcare Engineering, University College London, London, United Kingdom; ^6^Imperial College London, London, United Kingdom; ^7^Institute of Gerontology and Geriatrics, University of Perugia, Perugia, Italy; ^8^Institute of Clinical Medicine and Department of Neurology, University of Eastern Finland, Kuopio, Finland; ^9^Neurology, Neurocenter, Kuopio University Hospital, Kuopio, Finland; ^10^Department of Epidemiology and Biostatistics, VU University Medical Center, Amsterdam, Netherlands

**Keywords:** neurodegenerative diseases, classification, decision support, Alzheimer’s disease, frontotemporal lobar degeneration, vascular dementia, dementia with Lewy bodies

## Abstract

Clinical decision support systems (CDSSs) hold potential for the differential diagnosis of neurodegenerative diseases. We developed a novel CDSS, the PredictND tool, designed for differential diagnosis of different types of dementia. It combines information obtained from multiple diagnostic tests such as neuropsychological tests, MRI and cerebrospinal fluid samples. Here we evaluated how the classifier used in it performs in differentiating between controls with subjective cognitive decline, dementia due to Alzheimer’s disease, vascular dementia, frontotemporal lobar degeneration and dementia with Lewy bodies. We used the multiclass Disease State Index classifier, which is the classifier used by the PredictND tool, to differentiate between controls and patients with the four different types of dementia. The multiclass Disease State Index classifier is an extension of a previously developed two-class Disease State Index classifier. As the two-class Disease State Index classifier, the multiclass Disease State Index classifier also offers a visualization of its decision making process, which makes it especially suitable for medical decision support where interpretability of the results is highly important. A subset of the Amsterdam Dementia cohort, consisting of 504 patients (age 65 ± 8 years, 44% females) with data from neuropsychological tests, cerebrospinal fluid samples and both automatic and visual MRI quantifications, was used for the evaluation. The Disease State Index classifier was highly accurate in separating the five classes from each other (balanced accuracy 82.3%). Accuracy was highest for vascular dementia and lowest for dementia with Lewy bodies. For the 50% of patients for which the classifier was most confident on the classification the balanced accuracy was 93.6%. Data-driven CDSSs can be of aid in differential diagnosis in clinical practice. The decision support system tested in this study was highly accurate in separating the different dementias and controls from each other. In addition to the predicted class, it also provides a confidence measure for the classification.

## Introduction

Worldwide dementia affects over 47 million people and is one of the major causes of dependency and disability with huge social and economic impact (World Health Organization, 2016). Alzheimer’s disease (AD) is the most common cause of dementia and accounts for 60–70% of the dementia cases. At an older age, vascular dementia (VaD) and dementia with Lewy bodies (DLB) also frequently occur. Frontotemporal lobar degeneration (FTLD) is the second most prevalent type of dementia in patients with early onset. For therapeutical and research purposes, early and precise diagnosis is important (Román et al., 1993; Neary et al., 1998; McKeith et al., 2005; McKhann et al., 2011; Rascovsky et al., 2011; Snowden et al., 2011).

Cognitive profiles differ between dementia types showing primarily memory impairment in AD, visuospatial and executive dysfunction in DLB, delayed cognitive processing in VaD and mainly language, executive and behavioral dysfunction in FTD (Burrell and Piguet, 2015; Smits et al., 2015) although considerable overlap exists. Progress in biomarker development has provided new disease insights and improved accuracy of dementia diagnosis. This has led to an increasing role of biomarkers, such as those obtained from cerebrospinal fluid (CSF) measures and structural magnetic resonance imaging (MRI), in diagnostic criteria and guidelines (Román et al., 1993; McKhann et al., 2011; Rascovsky et al., 2011; McKeith et al., 2017). CSF biomarkers can provide evidence for the presence of beta amyloid 1-42 (AB42) accumulation and downstream neuronal dementia in AD [tau and tau phosphorylated at threonine 181 (p-tau)], while isolated elevation of tau may also be seen in FTD and intermediate concentrations of CSF biomarkers often occur in DLB and VaD (Mattsson et al., 2012; Schoonenboom et al., 2012; Blennow et al., 2015; Ewers et al., 2015; Llorens et al., 2016). On structural MRI, typical abnormalities for different causes of dementia have been described, such as hippocampal and parietal atrophy in AD, frontal-temporal atrophy in FTD, and profound white matter hyperintensities in VaD, whereas DLB present with unspecific mild generalized atrophy (Scheltens et al., 1997; Burton et al., 2009; Koedam et al., 2011; Rhodius-Meester et al., 2017). Also other measurement modalities which are not used in this study, such as 123I-FP-CIT SPECT imaging (Brigo et al., 2015), can provide useful information for the differential diagnosis.

Despite these advances, differential diagnosis of dementia in terms of accurately identifying the underlying etiology remains challenging. First, biomarkers for other types of dementia are less developed than those for AD and second, there is often overlap in underlying pathology and clinical presentation as most patients do not present in an archetypical fashion (Burton et al., 2009; Schoonenboom et al., 2012; Rivero-Santana et al., 2016; Simonsen et al., 2017). In addition, diagnostic guidelines remain relatively general and addresses one disease only. In reality, a clinician often faces a complex differential diagnostic task of simultaneously evaluating a range of potential diagnoses.

**FIGURE 1:**
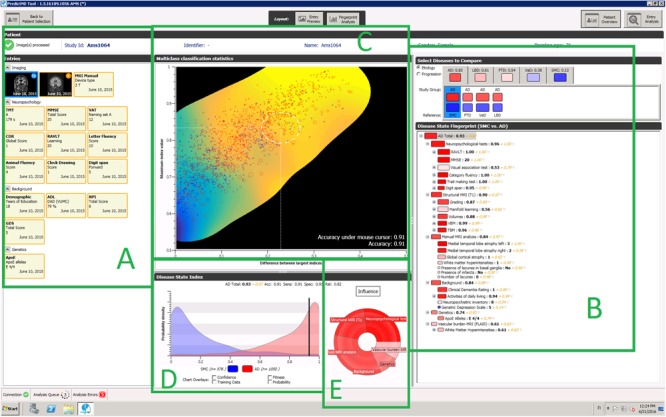
A screenshot of the PredictND tool, a CDSS for differential diagnosis of dementia. The tool contains for example: structured access to the raw data and to visualizations of the MRI analysis **(A)**, and a visualization of the hierarchical decision making of process of the DSI classifier **(B)**, visualization of the expected accuracy of the DSI classifier for this patient **(C)**, distribution of an individual biomarker for different diagnostic groups **(D)**, and visualization of relative influence of different measurement modalities to the DSI classifiers classification **(E)**.

Clinical decision support systems (CDSS) could provide a systematic and more objective way for helping clinicians in the complex reasoning related to differential diagnostics. Our previous work on the PredictAD CDSS tool was based on this concept, but the tool was developed to distinguish only between two classes, i.e., patients with AD vs. healthy controls, or stable vs. progressive MCI patients (Mattila et al., 2011, 2012a; Hall et al., 2015a; Rhodius-Meester et al., 2015). To reflect daily clinical practice more closely, we extended the tool to differential diagnosis of dementia. This extended tool is called the PredictND tool. In the tool data from a patient are compared with a large database of pre-existing patient measurements and corresponding diagnoses. This database forms the reference data for finding the disease patterns from data and measuring the patient’s similarity to these patterns (Mattila et al., 2011). The results of this statistical analysis and overview of available clinical data are then visualized to the users in a form that is easy to understand and can support their decision making. The user interface of the tool is shown in **Figure 1**. The classifier used by the tool is called the Disease State Index (DSI) classifier.

First CDSSs for differential diagnosis of dementia were presented already almost 30 years ago (Plugge et al., 1990, 1991). After this, multiple studies that are similar to our study presented here (in the sense that they have used automatic classification methods with similar measurement types for differential diagnosis of dementia) have been performed. Of the measurement modalities MRI has been the most common in these studies (Davatzikos et al., 2008; Klöppel et al., 2008; Muñoz-Ruiz et al., 2012; Raamana et al., 2014; Möller et al., 2016; Bron et al., 2017; Canu et al., 2017; Tu et al., 2017). Also neuropsychological tests (Diehl et al., 2005; Jiménez-Huete et al., 2014), CSF, MRI and FDG PET (Perani et al., 2016), and the combination of neuropsychological tests, MRI, CSF, SPECT, and genetic biomarkers (Muñoz-Ruiz et al., 2016) have been studied in this manner. As far as we know, besides our two earlier studies (Koikkalainen et al., 2016; Tong et al., 2017) no studies have addressed a similar five-class classification problem covering the most common forms of dementia. The earlier studies have at most addressed the classification of two dementia types (usually AD and FTLD) and controls, or three types of dementia (Jiménez-Huete et al., 2014).

The objective of this study is to evaluate the performance of the DSI classifier for classifying patients in differential diagnosis of dementias. In an earlier study we presented the MRI analysis methods used in the CDSS and evaluated the classification accuracy for differentiating between patients with AD, VaD, DLB, FTLD, and controls using only structural MRI data (Koikkalainen et al., 2016). In another study we introduced alternative MRI analysis methods, and tested different machine learning methods for the classification problem (Tong et al., 2017). Here we extend the first study (Koikkalainen et al., 2016) by evaluating the DSI classifier with a more comprehensive set of data, consisting of neuropsychological tests, CSF samples, and both automatic and visual MRI ratings.

## Data and Methods

### Patients and Clinical Assessment

We studied 504 patients from the Amsterdam Dementia Cohort who had visited the Alzheimer center between years 2004 and 2014 (van der Flier et al., 2014). We included subjects with a baseline diagnosis of AD, FTLD, DLB, or VaD. In addition, we included patients with a diagnosis of subjective cognitive decline (SCD) as controls. Patients were included if a neuropsychological test battery, MRI of brain, and CSF biomarkers were available. Subjects with SCD were selected to have a minimal follow up of 9 months during which they remained stable. The study was approved by the Medical Ethical Committee (Medisch Ethische Toetsingscommissie) of VUmc Medical Center. All patients have given written informed consent for their clinical data to be used for research purposes.

At baseline, all patients received a standardized and multidisciplinary workup, including medical history, physical, neurological and neuropsychological examination, MRI, laboratory test and lumbar puncture to collect CSF. Diagnoses were made in a multidisciplinary consensus meeting. Patients were diagnosed as having SCD when the cognitive complaints could not be confirmed by cognitive testing and criteria for MCI, dementia or other neurological or psychiatric disorder known to cause cognitive complaints were not met. Patients were diagnosed with probable AD using the criteria of the NINCDS-ADRDA (McKhann et al., 1984); all patients also met the core clinical criteria of the NIA-AA for probable AD (McKhann et al., 2011). FTLD was diagnosed using the Neary and Snowden criteria (Neary et al., 1998). Of the FTLD patients, 60 were diagnosed with behavioral variant frontotemporal dementia (bvFTD) additionally fulfilling the core criteria from Rasckovsky (Rascovsky et al., 2011), and 32 patients were diagnosed with a language variant (27 semantic dementia (SD) and 5 progressive non-fluent aphasia (PNFA)) additionally fulfilling the criteria of Gorno-Tempini (Gorno-Tempini et al., 2011). VaD was diagnosed using the NINDS-AIREN criteria (Román et al., 1993), and DLB using the McKeith criteria (McKeith et al., 1996, 2005).

A summary of the patient characteristics is presented in **Table 1**.

**Table 1:** Basic characteristics of the patients in different diagnostic categories.

	All	CN	AD	FTLD	DLB	VaD
*N*	504	118	223	92	47	24
Age	65 ± 8	61 ± 9^b,c,d,e^	66 ± 7^a,c^	63 ± 7^a,b,d,e^	68 ± 9^a,c^	69 ± 6^a,c^
Females	221 (44%)	45 (38%)^b,d^	120 (54%)^a,d^	41 (45%)^d^	6 (13%)^a,b,c,e^	9 (38%)^d^
MMSE	23 ± 5	28 ± 1^b,c,d,e^	21 ± 5^a,c,d,e^	24 ± 5^a,b^	23 ± 4^a,b^	24 ± 5^a,b^

*Statistically significant differences (*p* < 0.05) between the patient groups were studied using the Mann–Whitney *U* test for age and MMSE, and using Chi-squared test for the gender. Differences are marked as follows: ^*a*^statistically significantly different from control, ^*b*^statistically significantly different from AD, ^*c*^statistically significantly different from FTLD, ^*d*^statistically significantly different from DLB, ^*e*^statistically significantly different from VaD. MMSE, Mini Mental State Examination; CN, control.*

### Neuropsychological Tests

Cognitive functions were assessed with a standardized test battery consisting of the Mini Mental State Examination (MMSE) (Folstein et al., 1975), the Cambridge Examination for Mental Disorders of the Elderly (CAMCOG) (Derix et al., 1991) forward and backward conditions of Digit Span (Lindeboom and Matto, 1994), the Visual Association Test (VAT), the Rey Auditory Verbal Learning Test (RAVLT) (Saan and Deelman, 1986; Lindeboom et al., 2002), the Category Fluency Test (CFT) (animals) (Van der Elst et al., 2006), the Trail Making Test (TMT) (Reitan, 1958), the Frontal Assessment Battery (FAB) (Dubois et al., 2000), the Stroop test (Stroop, 1935) and the Rey figure copy test (Osterrieth, 1944). Depressive symptoms were assessed by the Geriatric Depression Scale (GDS) (Yesavage et al., 1982), behavioral and psychological symptoms by the Neuropsychiatric Inventory (NPI) (Cummings et al., 1994) and activities of daily living using the Disability Assessment for Dementia (DAD) (Gélinas et al., 1999).

All of the patients had MRI scans and CSF samples taken, but not all of the neuropsychological tests were performed in all patients. The proportions of patients for which each measurement was done are listed for each patient group in **Table 2**.

**Table 2:** Proportions of patients for which the different neuropsychological tests were done.

	All (*N* = 504) %	CN (*N* = 118) %	AD (*N* = 223) %	FTLD (*N* = 92) %	DLB (*N* = 47) %	VaD (*N* = 24) %
MMSE	100	100	100	100	100	100
CAMCOG	78	53	100	57	74	75
VAT	98	100	99	93	100	100
RAVLT	93	100	95	74	96	100
CFT	97	100	100	88	100	92
TMT	96	100	95	95	94	100
FAB	80	76	87	70	79	83
Stroop test	89	98	92	70	91	92
Rey figure copy	41	66	26	41	49	46
GDS	90	93	95	77	87	79
DAD	68	40	93	49	66	46
NPI	86	69	100	74	100	67

*MMSE, Mini Mental State Examination; CAMCOG, Cambridge Examination for Mental Disorders of the Elderly; VAT, Visual Association Test; RAVLT, Rey Auditory Verbal Learning Test; TMT, Trail Making Test; FAB, Frontal Assessment Battery; GDS, Geriatric Depression Scale; DAD, Disability Assessment for Dementia; NPI, Neuropsychiatric Inventory, CN, control.*

### Imaging

Subjects were scanned using either a 1.0 T (85 patients), 1.5 T (98 patients) or 3.0 T (321 patients) MR system. All scans were visually rated by a trained rater, and subsequently evaluated in a consensus meeting with an experienced neuroradiologist (van der Flier et al., 2014). All scans included a 3-dimensional T1-weighted gradient echo sequence and a fast fluid-attenuated inversion recovery (FLAIR) sequence. Visual rating of medial temporal lobe atrophy (MTA) was performed on coronal T1-weighted images according to the 5-point (0–4) Scheltens scale from the average score of the left and right sides (Scheltens et al., 1995). Global cortical atrophy (GCA) was assessed visually on axial FLAIR images (possible range of scores 0–3) (Pasquier et al., 1996). The degree of white matter hyperintensities severity was rated on axial FLAIR images using Fazekas’ scale (Fazekas et al., 1987). Lacunes were defined as T1-hypointense and T2-hyperintense CSF-like lesions surrounded by white matter or subcortical gray matter.

In addition to the visual quantifications the MRI images were quantified using six different automatic quantification methods in the PredictND tool. Multi-atlas segmentation based volumetry was used to measure the volume of 139 brain regions. Tensor and voxel based morphometry (TBM and VBM) techniques were used to quantify local shape-changes of the brain and the concentration of gray matter, respectively. Manifold learning and ROI based grading were used to measure the similarity of the MRI scans with a database of existing scans with known diagnoses. Vascular changes were quantified by a vascular burden measure based on segmentation of white matter hyperintensities, and cortical and lacunar infarcts. All these methods are described in more detail in Koikkalainen et al. (2016).

### Cerebrospinal Fluid

Cerebrospinal fluid analyses were performed at the Neurochemistry Laboratory at the department of Clinical Chemistry of the VU University Medical Center Amsterdam. CSF was obtained by lumbar puncture between the L3/L4 or L4/L5 intervertebral space by a 25-gauge needle and collected in polypropylene tubes. Within 2 h, the CSF was centrifuged at 1800 *g* for 10 min at 4°C, transferred to new polypropylene tubes, and stored at -20°C until biomarker analysis (within 2 months). Aβ1-42, total tau (t-tau) and tau phosphorylated at threonine 181 (p-tau) were measured with commercially available ELISAs (Innotest, Fujirebio, Ghent, Belgium).

### Classification Using the DSI Classifier

For classifying the patients we used a multiclass DSI classifier. The DSI classifier was originally designed for two-class classification problems (Mattila et al., 2011, 2012a). In addition to the class label it produces an index DSI(i,j) between zero and one describing the likelihood that the patient belongs to the class i when class j is the alternative option. A more detailed description of two-class DSI classifier is given in Appendix A in the Supplementary Material.

In order to convert the two-class DSI classifier into a multi-class classifier we computed a total index for each class. The total index DSI(i) for class i is the mean of two-class indices between class i and all other classes: $\mathrm{DSI}(i)=\frac{1}{\#\mathrm{classes}}\sum_{j\neq i}D\mathrm{SI}(i,j)$. Each patient is then classified to the class with the highest total index.

The total indices can also be used to quantify the classifiers confidence in the decision. The classification accuracy for patients with a very high maximum total index can be expected to be better, than for those patients for whom none of the classes receives a high total index.

In the training phase, we made two modifications to the training data. The modifications are based on *a priori* knowledge of usefulness of some of the MRI features. First, since there are no VaD specific structural changes, we have excluded the structural MRI features from all the pairwise classifications involving VaD. Second, when training the classifier for pairwise classification between classes A and B we only use TBM and VBM features that have been generated to separate the classes A and B. These modifications are the same as in our previous study (Koikkalainen et al., 2016). When the classifier is tested the same set of features is used for all patients, so that no information of class labels is given to the classifier.

### Classification Using RUSBoost

Because DSI treats each variable independently, it is incapable of learning classification rules in which the interpretation of one measurement depends on the value of another. It is likely that this type of connections exist between the variables, and a more complex classifier could, at least in theory, perform better classification by utilizing them. In order to test if a more complex classifier would outperform the DSI classifier, we have tested the five-class classification using also the RUSBoost algorithm (Seiffert et al., 2010). RUSBoost was in our earlier study the best classification method for this type of classification problem (Tong et al., 2017).

### Removal of Nuisance Variability

To reduce the effect of covariates such as age and gender to the classification, we normalized the features. This was done by fitting a multivariate linear regression model to the feature values of control group using the nuisance variables as explanatory variables. This model estimates the expected value of the feature given the nuisance variables, which is then subtracted from the actual feature values in order to obtain the normalized values (Koikkalainen et al., 2012).

The nuisance variables for which the measurement values were corrected for were: age, gender, education level, and MRI scanner type. The correction for MRI scanner type was done since we noticed systematic differences between MRI scans done with 1.0 T MRI device and other scanners; scanner type did not affect the classification accuracy using MRI (see Koikkalainen et al., 2016 for details). Education level was assessed using Verhage’s classification scale (Verhage, 1964).

For the neuropsychological tests, age, gender, and education level were used in the normalization; for the CSF biomarkers age and gender were used in the normalization; and for the automatic MRI quantifications age, gender and MRI scanner type were used in the normalization. The visual MRI ratings were not normalized for the nuisance variability.

### Performance Metrics

The simplest measure of classifier performance is the accuracy (Acc.), i.e., the proportion of correctly classified patients:

$$\begin{array}{c} \mathrm{Acc}.=\frac{\#\mathrm{correctly}\mathrm{classified}\mathrm{patients}}{\#\mathrm{all}\mathrm{patients}} \end{array}$$

This measure is, however, dependent on the number of cases in each group. If for example most patients in the data set belong to a single class, a classifier that always predicts this most frequent class will achieve an accuracy equal to the prevalence of this class, without using any information from patient measurements.

Therefore, we chose to use a multiclass extension of the balanced accuracy in addition to the accuracy to evaluate classifier performance (Brodersen et al., 2010). The balanced accuracy (Bal. acc.) is the mean of the sensitivities for each class, i.e., the proportion of patients belonging to each class that have been correctly classified:

$$\begin{array}{c} \mathrm{Bal}.\mathrm{acc}.=\frac{1}{\#\mathrm{classes}}\sum_{i=1}^{\#\;\mathrm{classes}}\frac{\#\mathrm{correctly}\mathrm{classified}\mathrm{patients}\mathrm{in}\mathrm{class}i}{\#\mathrm{patients}\mathrm{in}\mathrm{class}i} \end{array}$$

It is an estimate of the accuracy the classifier would achieve on a data set consisting of equal amount of patients in each class. The balanced accuracy is equal to $\frac{1}{\#\mathrm{classes}}$ if one assigns a class for a patient randomly, i.e., guesses the result. This means random guessing would yield an accuracy of 20% for the five-class classification problem in this study.

All performance measures were computed using 10-fold cross-validation.

## Results

### Classification Accuracies With Different Subsets of the Measurements

**Table 3** shows classification accuracies obtained for the five-class (AD, FTLD, DLB, VaD, and control) classification problem using all combinations of the four different data sources (neuropsychological tests, CSF biomarkers, visual MRI ratings and automatic MRI quantification) used in this study. The best single data source was the automatic MRI quantification (bal. acc. 66.1%). When all the data sources are used the balanced accuracy is 82.3%; and the classifier is most accurate for the vascular dementia cases (sensitivity 91.7%) and least accurate for the DLB cases (sensitivity 74.5%). The confusion matrix when using all the data sources is shown in **Table 4**. For a more detailed view of which data sources help in differentiating which classes from each other, the balanced accuracies for all possible two-class classification problems are shown in **Table 5**.

**Table 3:** Accuracy, balanced accuracy, and sensitivities [%] for all diagnostic groups, using different subsets of the data sources.

Feature set	Acc.	Bal. Acc.	Sens. CN	Sens. AD	Sens. FTLD	Sens. DLB	Sens. VaD
NP	62.3	57.3	83.1	61.9	48.9	46.8	45.8
CSF	51.2	40.6	40.7	72.2	35.9	12.8	41.7
VMRI	45.8	54.5	68.6	26.9	57.6	36.2	83.3
AMRI	66.3	66.1	78.8	63.7	68.5	31.9	87.5
NP and CSF	67.1	59.7	83.1	69.5	55.4	53.2	37.5
NP and VMRI	72.2	74.0	90.7	64.6	67.4	68.1	79.2
NP and AMRI	78.0	77.1	91.5	76.2	70.7	63.8	83.3
CSF and VMRI	63.9	62.3	63.6	69.5	58.7	40.4	79.2
CSF and AMRI	71.2	72.5	79.7	68.2	71.7	55.3	87.5
VMRI and AMRI	68.3	70.0	77.1	64.6	69.6	51.1	87.5
NP, CSF, and VMRI	75.8	73.7	89.0	73.1	71.7	68.1	66.7
NP, CSF, and AMRI	83.3	82.9	92.4	83.0	75.0	76.6	87.5
NP, VMRI, and AMRI	77.2	77.8	89.0	75.8	66.3	70.2	87.5
CSF, VMRI, and AMRI	71.0	74.5	78.0	67.3	67.4	68.1	91.7
All	81.5	82.3	89.0	80.3	76.1	74.5	91.7

*NP, neuropsychological tests; CSF, cerebrospinal fluid based biomarkers; VMRI, visual MRI ratings; AMRI, automatic MRI quantifications; Sens., sensitivity for each diagnostic group, i.e., the proportion of patients that are correctly classified in that group; CN, control.*

**Table 4:** Confusion matrix when all the measurements are used.

	CN	AD	FTLD	DLB	VaD
CN	105	1	4	7	1
AD	1	179	21	18	4
FTLD	5	6	70	8	3
DLB	1	7	2	35	2
VaD	0	1	0	1	22

*In the confusion matrix each row represents the clinical diagnosis and each column the diagnosis suggested by the classifier; the cells show the number patients in each category. CN, control.*

**Table 5:** Balanced accuracies [%] using different subsets of the data sources for all possible two-class classification problems.

Feature set	CN vs. AD	CN vs. FTLD	CN vs. DLB	CN vs. VaD	AD vs. FTLD	AD vs. DLB	AD vs. VaD	FTLD vs. DLB	FTLD vs. VaD	DLB vs. VaD
NP	96.3	87.8	96.0	94.1	74.4	78.4	77.6	74.7	73.9	65.3
CSF	87.6	62.5	60.5	64.8	79.1	79.4	75.4	58.1	66.5	51.6
VMRI	81.3	83.7	75.4	90.8	61.4	62.0	90.7	72.6	87.2	91.6
AMRI	91.1	89.6	79.2	96.2	80.3	71.9	94.3	80.7	95.2	95.8
NP and CSF	97.2	85.7	95.5	93.7	80.6	85.2	84.0	74.2	74.5	57.9
NP and VMRI	97.2	91.7	94.0	96.6	75.3	81.5	93.1	80.7	89.9	86.3
NP and AMRI	97.4	96.1	92.4	99.6	82.7	75.9	95.9	85.5	95.2	93.7
CSF and VMRI	91.6	86.8	74.1	90.8	80.4	78.8	92.5	75.2	89.3	89.5
CSF and AMRI	92.2	90.9	79.2	96.2	84.4	74.7	94.8	81.2	95.2	95.8
VMRI and AMRI	89.8	88.6	83.0	96.6	81.0	72.1	94.3	80.7	92.6	95.8
NP, CSF, and VMRI	96.5	91.8	93.0	97.5	84.0	85.4	91.5	80.1	89.9	86.3
NP, CSF, and AMRI	97.4	93.0	93.4	97.9	88.0	80.4	98.0	85.5	93.1	93.7
NP, VMRI, and AMRI	95.0	93.1	92.8	97.1	83.4	76.3	95.2	86.5	92.6	95.8
CSF, VMRI, and AMRI	91.5	90.4	82.4	96.6	81.1	78.2	94.3	82.3	92.6	95.8
All	96.8	92.1	92.4	97.1	87.2	79.9	95.5	86.5	93.1	95.8

*NP, neuropsychological tests; CSF, cerebrospinal fluid based biomarkers; VMRI, visual MRI ratings; AMRI, automatic MRI quantifications; CN, control.*

The neuropsychological test measurement values are not missing at random (see **Table 2**). The classifier could potentially exploit this information in the classification. In order to make sure the results are not biased, we tested the accuracy of the classification using a subset of the data without missing values, and found no major difference in classification accuracy to data with missing values. The details of this comparison can be found in Appendix B in the Supplementary Material.

In the comparison to RUSBoost, the DSI classifier outperforms it in overall accuracy: the balanced accuracy reached by RUSBoost is 75.5% when using all the measurements. However, RUSBoost performs better when some subsets of the data sources are used. Details of the comparison can be found in Appendix C in the Supplementary Material.

### Classification Accuracy vs. Confidence

**Table 6** shows how the classification accuracy increases when the cases for which the classifier is least confident are left out from the evaluation. The maximum of the total indices is used as the confidence measure. For example, if 50% of the cases were left out corresponding to the total index cut-off value 0.79, the accuracy was 95.2% and the balanced accuracy was 93.6%. Balanced accuracy is no longer computed when 75% of the cases are left out, since there are no DLB patients remaining in this subset.

**Table 6:** Classification accuracies when patients for which the classifier is least confident of the true class are left out.

Uncertain patients [%]	0.0	25.0	50.0	75.0
Total index cut-off	0.00	0.72	0.79	0.85
Accuracy [%]	81.5	91.0	95.2	99.2
Balanced accuracy [%]	82.3	89.4	93.6	N/A

*The columns show for different percentages of left out patients the DSI threshold used for rejecting uncertain patients, and the accuracy and balanced accuracy obtained when the patients are left out of the classification.*

Classification results and the percentage of patients left in each diagnostic group are shown in **Table 7**. The classifier is least confident on the classification of DLB patients, 76.6% of the DLB patients are left out from the 50% subset of patients for which the classifier is most confident on the correct class.

**Table 7:** Confusion matrix (on the left), and percentage of patients left out and sensitivity for each class (on the right), when 50% of the patients that the classifier is least confident of are left out.

	CN	AD	FTLD	DLB	VaD	Patients left out [%]	Sens. [%]
CN	79	0	0	0	0	33.1	100.0
AD	0	98	7	0	0	52.9	98.1
FTLD	0	2	40	0	1	53.3	92.9
DLB	0	1	0	9	1	76.6	81.8
VaD	0	0	0	0	13	45.8	100.0

## Discussion

In this study, we tested the classification accuracy of the DSI classifier for the differential diagnosis of dementia using different types of diagnostic tests: neuropsychological tests, CSF biomarkers, and automatic quantifications and visual ratings of MRI. Using all the diagnostic tests the system was highly accurate in separating the five classes (bal. acc. 82.3%).

When the role of different data sources is studied in detail (**Table 3**), automatic MRI quantification produced the best results. This implies patterns of atrophy are closely related to clinical presentation of the different types of dementia and that automatic image quantification is able to characterize images in a richer way than what can be done with current visual rating scales alone. Leaving automatic MRI quantification out had the largest impact on the classification accuracy; balanced accuracy dropped from 82.3% to 73.7%. The CSF based features perform the worst (bal. acc. 40.6%), which is seemingly in contrast with earlier studies on differential diagnoses and studies using a CDSS (Mattila et al., 2012b; Muñoz-Ruiz et al., 2013; Rhodius-Meester et al., 2015). However, all these former studies applied a two-class CDSS, comparing controls with AD, stable MCI with progressive MCI or AD with FTLD. In this study, CSF based biomarkers were highly useful when separating AD from other groups, but less so for separating between two non-AD groups. For example, classification accuracy for separating DLB cases from VaD cases using CSF biomarkers was close to 50%, i.e., equal to guessing the diagnosis (see **Table 5**). In the future, biomarkers specific for discriminating two types of non-AD dementias may help to further improve the diagnostic accuracy.

The results show also that all data sources (neuropsychology, MRI and CSF) are important: clearly the highest accuracy was obtained when all data sources were included. The best two data sources were neuropsychological tests combined with automatic MRI quantification, producing balanced accuracy of 77.1%. The balanced accuracy increased to 82.9% after adding the third data source.

In a comparison to a more complex classifier (RUSBoost) the DSI classifier performs favorably reaching a higher accuracy when all data sources are used (balanced accuracy 82.3% vs. 75.5%), but RUSBoost outperforms DSI using some subsets of the data sources such neuropsychological tests and CSF. As the DSI classifier also has other advantages such as interpretability of the results, we feel that it is more suitable classifier for decision support for this particular case. It is possible that a combination of a complex machine learning method and a transparent classifier such as DSI could offer the optimal tradeoff between accuracy and interpretability of results.

Both the DSI classifier and RUSBoost obtained a slightly higher classification accuracy when the visual MRI ratings are left out, when compared to classification using all measurements. The balanced accuracy increases from 82.3 to 82.9% for DSI classifier, and from 75.5 to 77.0% for RUSBoost. The difference is so small for both classifiers, that it is not possible to say whether the visual MRI ratings actually decrease the classification performance. It is also possible that the difference is coincidental, or based on a peculiarity in this specific data set. Therefore, we report the classification accuracies using all measurements as the overall accuracy for both classifiers.

Comparison of the classification results obtained in this study to other studies is not straightforward as the study populations and measurements used in the classification vary across studies, and most studies report results only for pairwise comparison of two patient groups. Only studies in which the five-class classification has been done are our two previous studies (Koikkalainen et al., 2016; Tong et al., 2017). The classification accuracy for the five-class problem is higher in this study than in either of those studies [82.3% vs. 69.3% in Tong et al. (2017) and 70.6% in Koikkalainen et al. (2016)], but here a wider set of measurements is used. We also tested the RUSBoost algorithm which provided best results in Tong et al. (2017), and showed that DSI classifier produced comparable results. The classification results obtained for the pairwise classifications in this study are similar to results previously reported in the literature. For the pairwise classification problem of separating dementia patients from controls, even accuracies of 100% have been reported (Davatzikos et al., 2008; Raamana et al., 2014), the balanced accuracies in this study varied from 92.4 to 96.8% depending on the dementia type. For the pairwise classification of different dementia groups the classification accuracies in earlier studies are much lower than for dementia patients vs. control classification. For AD vs. FTLD (Klöppel et al., 2008) reached a balanced accuracy of 89% (87.2% in this study). For AD vs. DLB (Jiménez-Huete et al., 2014) reached a balanced accuracy of 86% (79.9% in this study), and 62% for DLB vs. FTLD (86.5% in this study). These results are, however, highly dependent on the patient populations and measurement modalities used. A thorough comparison of the different pairwise classification results, which takes into account these issues, is beyond the scope of this study.

An essential question is what a balanced accuracy of over 80% for the five-class classification means clinically. Multiple issues must be taken into account when considering the answer. (1) The ground truth diagnosis used in this study was the clinical diagnosis. The agreement between clinical diagnosis and post-mortem neuropathological diagnosis has been reported to be 70–90% in dementias (Kazee et al., 1993; Lim et al., 1999; Jellinger, 2002), being comparable with the accuracy obtained in this study. Although neuropathological analyses are commonly considered as a ground truth, they are also imperfect and not without challenges (Scheltens and Rockwood, 2011). (2) Even if the accuracy were known exactly, one still needs to decide what level of accuracy is acceptable in clinical practice. Cost-efficiency analysis should be used to help answer this question in future studies. (3) One constraint of the study was that the ground truth diagnosis was a single disease although we know that 20–40% dementia patients have mixed dementia (Zekry et al., 2002), i.e., more than one underlying pathology. It is possible that our database contained cases for which the classifier found the best fit for another underlying disease which was not defined as the ground truth diagnosis in the database. Future studies should analyze whether a good match to two diseases could be an indication of mixed dementia, not just of the classifier’s difficulty to define the correct disease.

The classification method used in this study offers also a confidence estimate for the classification, which can be used to estimate how likely it is that the classification suggested by the classifier is correct. The classifier is considerably more accurate for those cases for which it is more confident of the correct class, i.e., DSI is high, (balanced accuracy of 93.6% for the most confident 50% vs. 82.3% for all patients). However, many of the patients for which the tool was not confident of correct class, are likely to be those patients for which a decision support tool would be most critically needed. The value of the tool among the cases which are most challenging to the clinician could be evaluated in a future study. In this study the classification was least accurate in FTLD (sensitivity 76.1%) and DLB (sensitivity 74.5%), both being disorders that can be hard to recognize. In these cases, a clinician could use the tool to narrow down the differential diagnosis. The tool could also aid the clinician by presenting the available data in a manner, which allows an easy overview of all the available measurements, and how they contribute to the classification (see **Figure 1**). The sensitivity of the tool might be increased by adding more disease-specific features, such as the presence of parkinsonism or hallucinations for DLB, or presence of changes in personality in bvFTD. Another challenge is the broad spectrum of FTLD; in this study we included patients with bvFTD, SD and PNFA. The language variants are likely to be easier to classify due to highly specific pattern of atrophy, while the differentiation between bvFTD and AD is far more challenging.

In a real-world decision-making scenario all of the options are usually not equally likely *a priori*, e.g., in the general population AD is more prevalent than other dementia types. In addition, prevalence of the different types of dementia may differ according to setting, with other types of dementia being very rare in a GP’s office, still quite rare in a local memory clinic, but relatively common in a tertiary referral setting. Positive predictive value and negative predictive value depend on the prevalence of disease; therefore, it is very important to take into account the *a priori* information on relative prevalence of diseases in the setting where the tool would be used. As there is no objectively right choice for the prior probabilities, we assumed in this study all diagnoses to be equally likely *a priori*. This assumption makes interpretation of the results easier, as the classifier uses only the measurement values to make the decisions and is not relying on assumptions about the prevalence of different conditions. Different prevalences of the diseases can be taken into account when developing the tool, e.g., by giving higher weight to more prevalent classes when computing the class indices from the pairwise comparisons.

In this study, not all neuropsychological tests were performed for every patient (**Table 2**). On one hand, this represents a realistic clinical scenario, all tests are not performed to every patient in real-life either. On the other hand this can affect for example the analysis of the importance of different data sources. Excluding patients with any missing values is a solution to this problem, but in this study, it would have meant leaving out a significant amount of patients. Therefore, we chose to perform the analysis using also patients with missing data. As our comparison (Appendix B in the Supplementary Material) shows, this does not have a large impact on the classification accuracy obtained by neuropsychological test data.

To support the clinician in daily practice the PredictND tool should be applicable in other clinical settings as well. Here the tool uses a large dataset from one tertiary memory clinic. The DSI classifier is a data-driven method that can use all available information from a specific population to fit the classification model. It is preferably trained on center-specific data, but we have shown that it can also be successfully trained using other available datasets assuming they are sufficiently similar (Hall et al., 2015b). This means the tool can also be implemented in daily practice in smaller clinics, possibly using a less extensive evaluation, and is not limited to be used in specialized centers.

## Conclusion

In conclusion, we evaluated the accuracy of the classification method used in the PredictND tool, which integrates information from multiple data sources, in differential diagnosis of dementia. The study was conducted using a large standardized data set from a tertiary memory clinic.

The results show that CDSSs can be of use in the differential diagnosis of dementias. The DSI classifier is highly accurate in classifying the patients to the five diagnostic groups achieving a balanced accuracy of 82.3%. It also offers a confidence measure for the classification, which can be used to select patients for which the classification accuracy is even higher.

To evaluate the contribution of the tool to daily clinical practice, the PredictND tool is currently tested in a prospective study in several European memory clinics. In this prospective study we collect a data set containing a complete set of data (neuropsychological tests, CSF sample, genetic biomarkers and MRI) for all patients. The data collection methods have also been harmonized across the different memory clinics as much as possible without interfering with the clinical work.

## Author Contributions

AT contributed the analysis and interpretation of data, and drafted and revised the manuscript for intellectual content. HR-M, MBr, FB, AL, TK, PS, CT, MBa, HS, AR, GW, SH, PM, and WvdF contributed to the study concept and design, and revised the manuscript for intellectual content. JK, TT, RG, AS, CL, DR, and JL contributed to the analysis and interpretation of data, and revised the manuscript for intellectual content.

## Conflict of Interest Statement

CT serves on the advisory board of Fujirebio and Roche, received research consumables from Euroimmun, IBL, Fujirebio, Invitrogen and Meso Scale Discovery, and performed contract research for IBL, Shire, Boehringer, Roche and Probiodrug; and received grants from the European Commission, the Dutch Research Council (ZonMW), Association of Frontotemporal Dementia/Alzheimer’s Drug Discovery Foundation, ISAO and the Alzheimer’s Drug Discovery Foundation. CT has received research consumables from Euroimmun, IBL, Fujirebio, Invitrogen and Meso Scale Discovery, and performed contract research for IBL, Shire, Boehringer, Roche and Probiodrug. CT has received lecture fees from Roche and Axon Neurosciences. JL and JK are shareholders and founders of Combinostics Ltd. They are also inventors in the following patents relevant to the subject of the study, for which Combinostics Ltd owns the IPR: (1) J. Koikkalainen and J. Lotjonen. A method for inferring the state of a system, US7,840,510 B2, PCT/FI2007/050277. (2) J. Lotjonen, J. Koikkalainen, and J. Mattila. State Inference in a heterogeneous system, PCT/FI2010/050545. FI20125177. The other authors declare that the research was conducted in the absence of any commercial or financial relationships that could be construed as a potential conflict of interest.
