# Supplementary material for: Data-Driven Differential Diagnosis of Dementia Using Multiclass Disease State Index Classifier
Source: Front Aging Neurosci. 2018 Apr 25;10:111. doi: 10.3389/fnagi.2018.00111 (PMC5996907; doi:10.3389/fnagi.2018.00111)
Supplement: Supplementary file 1 [file Data_Sheet_1.docx]

Supplementary Material

Data-Driven Differential Diagnosis of

Dementia Using Multiclass Disease

State Index Classifier

Antti Tolonen^1*^, Hanneke F.M. Rhodius-Meester^2^, Marie Bruun^3^, Juha Koikkalainen^4^, Frederik Barkhof^2, 5^, Afina W. Lemstra^2^, Teddy Koene^2^, Philip Scheltens^2^, Charlotte E. Teunissen^2^, Tong Tong^6^, Ricardo Guerrero^6^, Andreas Schuh^6^, Christian Ledig^6^, Marta Baroni^7^, Daniel Rueckert^6^, Hilkka Soininen^8, 9^, Anne M. Remes^8, 9^, Gunhild Waldemar^3^, Steen Gregers Hasselbalch^3^, Patrizia Mecocci^7^, Wiesje M. van der Flier^2, 10^, Jyrki Lötjönen^4^

^1^VTT Technical Research Centre of Finland, Tampere, Finland

^2^Alzheimer Center, Department of Neurology, VU University Medical Centre, Amsterdam Neuroscience, Amsterdam, the Netherlands

^3^Danish Dementia Research Centre, Rigshospitalet, Copenhagen, Denmark

^4^Combinostics Ltd., Tampere, Finland

^5^Institutes of Neurology and Healthcare Engineering, University College London, London, United Kingdom

^6^Imperial College London, London, United Kingdom

^7^Institute of Gerontology and Geriatrics, University of Perugia, Perugia, Italy

^8^Institute of Clinical Medicine, Neurology, University of Eastern Finland, Kuopio, Finland

^9^Neurocenter, Neurology, Kuopio University Hospital, Kuopio, Finland

^10^Department of Epidemiology and Biostatistics, VU University Medical Centre, Amsterdam, the Netherlands

*** Correspondence:** Antti Tolonen: antti.tolonen@vtt.fi

**Appendix A. Description of the two-class Disease State Index classifier**

The two-class DSI classifier compares patient's feature values to the feature values of patients for which the class is known. The comparison is done using the distributions of the feature values in both classes, and it is evaluated to which distribution the patient feature better fits. The classifier computes two statistics for each feature, fitness and relevance.

If the feature value is on average smaller in class 0 than in class 1, the fitness for the feature is computed as:

$$fitness\left( x \right)= \frac{L_{1}(x)}{L_{1}\left( x \right)+ R_{0}(x)}$$

Where $x$ is the value of the feature for the patient,$L_{1}(x)$ is the left integral of probability density function for class 1 and $R_{0}(x)$ is the left integral of probability density function for class 0. If the patient’s feature value is higher than any of the patients in class 0 have, the fitness value is one. On the other hand, a value of zero indicates that none of the patients in class 1 have that low feature values.

Relevance for a feature is computed as:

$$relevance=max\{0, L_{0}\left( x^{*} \right)+R_{1}\left( x^{*} \right)-1\}$$

Where $x^{*}$is the feature value for which $fitness\left( x^{*} \right)=0.5$, $L_{0}(x)$ is the left integral of probability density function for class 0 and $R_{1}(x)$ is the left integral of probability density function for class 1. The relevance is equal to the sum of sensitivity and specificity minus one using $x^{*}$ as the cut-off threshold for classification.

The DSI for a group of features is computed as a relevance-weighted sum of fitness of each feature:

$$DSI\left( x_{1}, x_{2}, \cdots, x_{n} \right)=\frac{\sum_{i=1}^{n} relevance\left( i \right)fitness(x_{i})}{\sum_{i=1}^{n} relevance(i)}$$

The DSI for a group of features can also computed so that they are organized into a tree-like hierarchy of features; e.g. all CSF features from one branch of the tree and all the MRI based features from another branch of tree, both of which can have their own sub-branches such as TBM based features in the case of MRI. In this case the DSI is computed for each branch by first computing the DSI for all the sub-branches, and using these sub-branch DSIs as new features when computing the DSI for the branch.

Using these hierarchies has two benefits over combining all the features in a single step when computing the DSI: 1) It provides useful intermediate results which tell the users of the importance of subgroups of the features and to which diagnosis the features in those subgroups point to. 2) It ensures that measurement modalities that produce huge number of features, such as automatic MRI analysis, do not occlude measurement modalities such as CSF in the decision making of the classifier by the sheer number of features they provide.

The hierarchy used in the PredictND tool is built based on a grouping of features by domain experts. An illustration of the hierarchy is shown in Figure A-1.

**
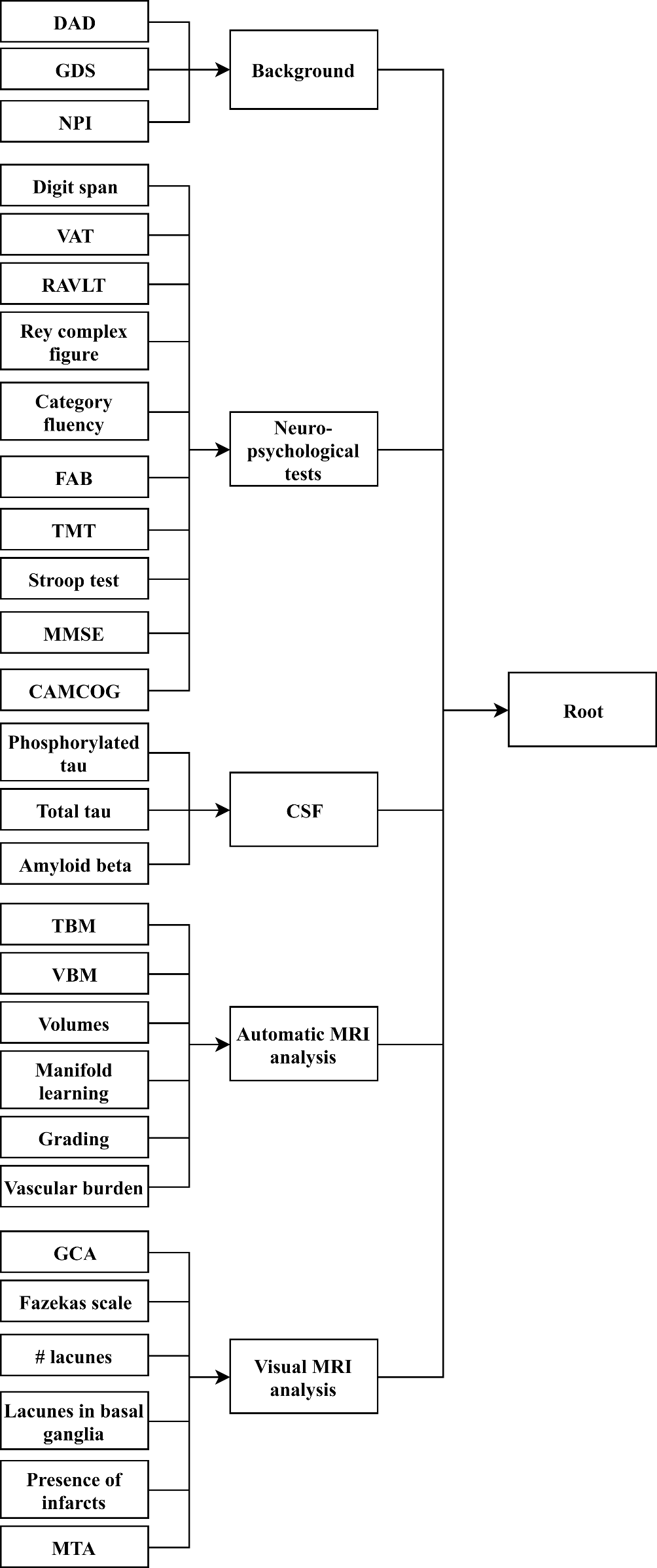
**

**Figure A-1: An illustration of the feature hierarchy used by the DSI classifier in the PredictND tool. The DSI classifier gives the final classification based on hierarchical evaluation of the tree from leafs (individual measurements) to the root node. The user of the PredictND tool can exclude nodes in the tree, in case he/she wants to use only a subset of the measurements in the classification.**

# Appendix B. Effect of missing data to the classification accuracy

Not all of the patients have all the measurements done, and different patient groups have different amounts of missing measurements. In principle this is information that the classifier could exploit while making the classification, e.g. if the patient is missing the Rey figure test score it is more likely that the patient belongs to AD group, where only 26 % of patients have the test score, than to the SCD group, where 66 % of the patients have the test score. In order to see that the classification results are not biased by these patterns in the missing data, we tested the classifier also on a subset of the data where all the patients have all measurements available. In this comparison only the neuropsychological tests are used for classification since they are the only measurement modality with significant number of missing values. In order to keep the size of this data set large enough we left out the Rey complex figure, DAD and CAMCOG tests from the data set, since these are measurements with the highest number of missing values. The performance of the classifier on the subset of the patients with all measurements (acc. 64.7 %, bal. acc. 48.0 %) is very similar to the performance on all patients (acc. 61.7 %, bal. acc. 52.2 %). More detailed results are shown in Table B-1.

**Table B-1. Confusion matrices (on the left) and sensitivities (on the right) for the five-class classification problem using data without missing values (Full) and using data with missing values (Sparse). In the confusion matrix each row represents the clinical diagnosis and each column the diagnosis suggested by the classifier; the cells show the number patients in each category. Only neuropsychological tests were used when computing the results. Abbreviations used: Sens. = sensitivity for each diagnostic group, i.e. the proportion of patients that are correctly classified in that group, CN = control.**

| Full | CN | AD | FTLD | DLB | VaD | Sens. [%] |
| --- | --- | --- | --- | --- | --- | --- |
| CN | 39 | 0 | 5 | 1 | 1 | 84.8 |
| AD | 2 | 53 | 24 | 8 | 9 | 55.2 |
| FTLD | 8 | 1 | 20 | 0 | 3 | 62.5 |
| DLB | 0 | 2 | 3 | 3 | 0 | 37.5 |
| VaD | 1 | 0 | 1 | 2 | 1 | 20.0 |
|  |  |  |  |  |  |  |
| Sparse | CN | AD | FTLD | DLB | VaD | Sens. [%] |
| CN | 99 | 1 | 13 | 1 | 4 | 83.9 |
| AD | 3 | 139 | 35 | 24 | 22 | 62.3 |
| FTLD | 10 | 15 | 47 | 7 | 13 | 51.1 |
| DLB | 1 | 8 | 6 | 24 | 8 | 51.1 |
| VaD | 1 | 2 | 1 | 9 | 11 | 45.8 |

# Appendix C. Comparison of DSI and RUSBoost

As DSI treats each variable independently, it is incapable of learning classification rules in which the interpretation of one measurement depends on the value of another. It is likely that this type of connections exist between the variables and a more complex classifier could in theory perform better classification by utilizing them, on the other hand a more complex classifier requires more data in order to find robust classification rules. This trade-off between complexity and robustness is known as in machine learning as bias-variance tradeoff (Theodoridis and Koutroumbas, 2009). In order to test if a more complex classifier would outperform the DSI classifier, we have tested the five-class classification using also RUSBoost algorithm (Seiffert et al., 2010). We have run the RUSBoost for 500 iterations and used a decision tree as the weak learner.

The results of the DSI classifier for different subsets of data sources are shown in Table C-1 (this table is identical to Table 4 in the manuscript and reproduced here only to make the Appendix C self-contained). The results of the RUSBoost for different subsets of data sources are shown in Table C-2, and differences between DSI and RUSBoost are shown in Table C-3.

When training the RUSBoost we have not removed any MRI imaging biomarkers in the training phase as was done for the DSI classifier. As the RUSBoost does not internally perform pairwise comparisons between the classes, the removal of the features severely reduces its performance for MRI imaging biomarkers. Using the same training data as the DSI classifier, the accuracy and balanced accuracy for automatic MRI quantifications were 49.4 % and 47.1 % respectively; compared to 67.6 % and 66.6 % using the unmodified training data. In order to make the comparison between the classifiers fair, we have used the unmodified training data for the RUSBoost.

**Table C-1 DSI’s accuracy, balanced accuracy, and sensitivities [%] for all diagnostic groups, using different subsets of the data sources. Abbreviations used: NP = neuropsychological tests, CSF = cerebrospinal fluid based biomarkers, VMRI = visual MRI ratings, AMRI = automatic MRI quantifications. Sens. = sensitivity for each diagnostic group, i.e. the proportion of patients that are correctly classified in that group, CN = control.**

| DSI classifier | | | | | | | |
| --- | --- | --- | --- | --- | --- | --- | --- |
| Feature set | Acc. | Bal. Acc. | Sens. CN | Sens. AD | Sens. FTLD | Sens. DLB | Sens. VaD |
| NP | 62.3 | 57.3 | 83.1 | 61.9 | 48.9 | 46.8 | 45.8 |
| CSF | 51.2 | 40.6 | 40.7 | 72.2 | 35.9 | 12.8 | 41.7 |
| VMRI | 45.8 | 54.5 | 68.6 | 26.9 | 57.6 | 36.2 | 83.3 |
| AMRI | 66.3 | 66.1 | 78.8 | 63.7 | 68.5 | 31.9 | 87.5 |
| NP and CSF | 67.1 | 59.7 | 83.1 | 69.5 | 55.4 | 53.2 | 37.5 |
| NP and VMRI | 72.2 | 74.0 | 90.7 | 64.6 | 67.4 | 68.1 | 79.2 |
| NP and AMRI | 78.0 | 77.1 | 91.5 | 76.2 | 70.7 | 63.8 | 83.3 |
| CSF and VMRI | 63.9 | 62.3 | 63.6 | 69.5 | 58.7 | 40.4 | 79.2 |
| CSF and AMRI | 71.2 | 72.5 | 79.7 | 68.2 | 71.7 | 55.3 | 87.5 |
| VMRI and AMRI | 68.3 | 70.0 | 77.1 | 64.6 | 69.6 | 51.1 | 87.5 |
| NP, CSF and VMRI | 75.8 | 73.7 | 89.0 | 73.1 | 71.7 | 68.1 | 66.7 |
| NP, CSF and AMRI | 83.3 | 82.9 | 92.4 | 83.0 | 75.0 | 76.6 | 87.5 |
| NP, VMRI and AMRI | 77.2 | 77.8 | 89.0 | 75.8 | 66.3 | 70.2 | 87.5 |
| CSF, VMRI and AMRI | 71.0 | 74.5 | 78.0 | 67.3 | 67.4 | 68.1 | 91.7 |
| All | 81.5 | 82.3 | 89.0 | 80.3 | 76.1 | 74.5 | 91.7 |

**Table C-2 RUSBoost’s accuracy, balanced accuracy, and sensitivities [%] for all diagnostic groups, using different subsets of the data sources. Abbreviations used: NP = neuropsychological tests, CSF = cerebrospinal fluid based biomarkers, VMRI = visual MRI ratings, AMRI = automatic MRI quantifications. Sens. = sensitivity for each diagnostic group, i.e. the proportion of patients that are correctly classified in that group, CN = control.**

| RF classifier | | | | | | | |
| --- | --- | --- | --- | --- | --- | --- | --- |
| Feature set | Acc. | Bal. Acc. | Sens. CN | Sens. AD | Sens. FTLD | Sens. DLB | Sens. VaD |
| NP | 70.6 | 59.5 | 99.2 | 83.9 | 17.4 | 55.3 | 41.7 |
| CSF | 63.3 | 48.6 | 68.6 | 85.2 | 32.6 | 19.1 | 37.5 |
| VMRI | 40.7 | 52.6 | 73.7 | 16.6 | 42.4 | 46.8 | 83.3 |
| AMRI | 67.7 | 66.6 | 83.1 | 67.7 | 63.0 | 23.4 | 95.8 |
| NP and CSF | 74.8 | 62.2 | 97.5 | 91.5 | 25.0 | 51.1 | 45.8 |
| NP and VMRI | 70.8 | 65.8 | 99.2 | 82.1 | 10.9 | 61.7 | 75.0 |
| NP and AMRI | 79.6 | 74.4 | 97.5 | 87.0 | 57.6 | 34.0 | 95.8 |
| CSF and VMRI | 68.5 | 63.0 | 77.1 | 80.3 | 46.7 | 23.4 | 87.5 |
| CSF and AMRI | 71.0 | 68.2 | 83.1 | 73.1 | 69.6 | 23.4 | 91.7 |
| VMRI and AMRI | 69.6 | 67.4 | 81.4 | 71.3 | 67.4 | 25.5 | 91.7 |
| NP, CSF and VMRI | 77.2 | 71.2 | 99.2 | 89.7 | 21.7 | 74.5 | 70.8 |
| NP, CSF and AMRI | 81.5 | 77.0 | 98.3 | 87.4 | 63.0 | 40.4 | 95.8 |
| NP, VMRI and AMRI | 78.2 | 73.1 | 98.3 | 85.2 | 54.3 | 31.9 | 95.8 |
| CSF, VMRI and AMRI | 72.2 | 68.7 | 82.2 | 76.7 | 67.4 | 25.5 | 91.7 |
| All | 80.8 | 75.5 | 98.3 | 88.3 | 58.7 | 36.2 | 95.8 |

**Table C-3 Difference between DSI’s and RUSBoost’s accuracies, balanced accuracies, and sensitivities [%] for all diagnostic groups, using different subsets of the data sources. Abbreviations used: NP = neuropsychological tests, CSF = cerebrospinal fluid based biomarkers, VMRI = visual MRI ratings, AMRI = automatic MRI quantifications. Sens. = sensitivity for each diagnostic group, i.e. the proportion of patients that are correctly classified in that group, CN = control.**

| Difference: DSI classifier - RUSBoost | | | | | | | |
| --- | --- | --- | --- | --- | --- | --- | --- |
| Feature set | Acc. | Bal. Acc. | Sens. CN | Sens. AD | Sens. FTLD | Sens. DLB | Sens. VaD |
| NP | -8.3 | -2.2 | -16.1 | -22.0 | 31.5 | -8.5 | 4.2 |
| CSF | -12.1 | -8.0 | -28.0 | -13.0 | 3.3 | -6.4 | 4.2 |
| VMRI | 5.2 | 2.0 | -5.1 | 10.3 | 15.2 | -10.6 | 0.0 |
| AMRI | -1.4 | -0.5 | -4.2 | -4.0 | 5.4 | 8.5 | -8.3 |
| NP and CSF | -7.7 | -2.4 | -14.4 | -22.0 | 30.4 | 2.1 | -8.3 |
| NP and VMRI | 1.4 | 8.2 | -8.5 | -17.5 | 56.5 | 6.4 | 4.2 |
| NP and AMRI | -1.6 | 2.7 | -5.9 | -10.8 | 13.0 | 29.8 | -12.5 |
| CSF and VMRI | -4.6 | -0.7 | -13.6 | -10.8 | 12.0 | 17.0 | -8.3 |
| CSF and AMRI | 0.2 | 4.3 | -3.4 | -4.9 | 2.2 | 31.9 | -4.2 |
| VMRI and AMRI | -1.4 | 2.5 | -4.2 | -6.7 | 2.2 | 25.5 | -4.2 |
| NP, CSF and VMRI | -1.4 | 2.5 | -10.2 | -16.6 | 50.0 | -6.4 | -4.2 |
| NP, CSF and AMRI | 1.8 | 5.9 | -5.9 | -4.5 | 12.0 | 36.2 | -8.3 |
| NP, VMRI and AMRI | -1.0 | 4.6 | -9.3 | -9.4 | 12.0 | 38.3 | -8.3 |
| CSF, VMRI and AMRI | -1.2 | 5.8 | -4.2 | -9.4 | 0.0 | 42.6 | 0.0 |
| All | 0.8 | 6.8 | -9.3 | -8.1 | 17.4 | 38.3 | -4.2 |

# References

Seiffert, C., Khoshgoftaar, T. M., Van Hulse, J., and Napolitano, A. (2010). RUSBoost: A hybrid approach to alleviating class imbalance. *IEEE Trans. Syst. Man, Cybern. Part ASystems Humans* 40, 185–197. doi:10.1109/TSMCA.2009.2029559.

Theodoridis, S., and Koutroumbas, K. (2009). *Pattern Recognition*. 4th ed. Academic Press.
